# Hemin and Zinc Protoporphyrin IX Affect Granisetron Constipating Effects In Vitro and In Vivo

**DOI:** 10.1155/2013/612037

**Published:** 2013-06-20

**Authors:** Addolorata Zigrino, Valentina Leo, Giuseppe Renna, Monica Montagnani, Maria Antonietta De Salvia

**Affiliations:** Department of Biomedical Sciences and Human Oncology, Pharmacology Section, Medical School, University of Bari “Aldo Moro”, Piazza Giulio Cesare, 70124 Bari, Italy

## Abstract

Granisetron is a 5-HT_3_ receptors antagonist used in the management of emesis associated with anticancer chemotherapy. It affects intestinal motility with constipating effect. Since the pathway heme oxygenase/carbon monoxide (HO/CO) is involved in gastrointestinal motility, we evaluated the possible interplay between granisetron and agents affecting HO/CO pathways such as zinc protoporphyrin IX (ZnPPIX), an HO inhibitor, or hemin, an HO-1 inducer. ZnPPIX (10 *µ*M) or hemin (10 *µ*M), but not granisetron (0.1, 0.3, 1 *µ*M), affected spontaneous basal activity recorded in rat duodenal strips, in noncholinergic nonadrenergic conditions. Granisetron restored spontaneous basal activity after ZnPPIX, but not after hemin. ZnPPIX decreased and hemin increased the inhibition of activity after electrical field stimulation (EFS), but they did not affect the contraction that follows the relaxation induced by EFS called off contraction. Granisetron did not alter the response to EFS per se but abolished both ZnPPIX and hemin effect when coadministered. In vivo study showed constipating effect of granisetron (25, 50, 75 *µ*g/kg/sc) but no effect of either ZnPPIX (50 *µ*g/kg/i.p.) or hemin (50 *µ*M/kg/i.p.). When coadministered, granisetron effect was abolished by ZnPPIX and increased by hemin. Specimens from rats treated in vivo with hemin (50 *µ*M/kg/i.p.) showed increased HO-1 protein levels. In conclusion, granisetron seems to interact with agents affecting HO/CO pathway both in vitro and in vivo.

## 1. Introduction

It is well known that, in mammals, a very high amount of 5-hydroxytryptamine (5-HT or serotonin) is synthesized within the gastrointestinal tract. In particular, 5-HT is mainly present in enterochromaffin cells of the mucosa and less represented in the neurons of the enteric nervous system, exerting its physiological effects acting on several 5-HT receptors. Therefore, a growing number of studies have been undertaken both in humans and using animal models to investigate the involvement of 5-HT in gastrointestinal disorders. Those studies have led to drugs acting at enteric 5-HT receptor subtypes, namely, 5-HT_3_ receptors, that have significantly improved the management of emesis associated with anticancer chemotherapy [1]. In fact, both first (dolasetron, granisetron, ondansetron, and tropisetron) and second (palonosetron) generation of 5-HT_3_ receptor antagonists are indicated by current guidelines as one of the pharmacologic interventions for acute and delayed nausea and vomiting associated with moderately and highly emetogenic chemotherapy, the other being dexamethasone and the NK_1_ receptor antagonist [2, 3]. 

Moreover, reduced visceral pain perception and colonic motor response to a meal together with reduced fluid secretion and colonic transit seem to follow blockade of enteric 5-HT_3_ receptors [4]. Those findings suggested that 5-HT_3_ receptor antagonists could be used in the treatment of irritable bowel syndrome (IBS): in fact, alosetron was approved for the treatment of women affected by diarrhea-predominant IBS, although its use was affected by a severe side effect such as ischemic colitis [5].

However, 5-HT is only one of the several neurotransmitters involved in the complex system of immune, sensory, secretion, and motility functions of gastrointestinal tract.

Recently the so-called gasotransmitters which act as neurally released transmitters, that is, nitric oxide (NO), carbon monoxide (CO), and hydrogen sulfide (H_2_S) have been proposed as neurotransmitters involved in some gastrointestinal functions [6].

In particular, CO, one of the by-products of heme oxygenase (HO), was shown to be a noncholinergic nonadrenergic (NANC) inhibitory neurotransmitter in the gastrointestinal tract [7]. In fact, CO activates soluble guanylyl cyclase which causes an increase of cGMP levels. This effect leads to activation of cGMP-dependent protein kinase that acts on several mechanisms that regulate intracellular levels of calcium in the smooth muscle cells resulting in relaxation [8]. Furthermore, CO seems to have anti-inflammatory and immunomodulating properties in the gastrointestinal tract [9]. 

Several studies have underlined the role of CO and/or HO induction in gastrointestinal diseases in animal models. Our research group has recently found the involvement of HO/CO pathway in conditions such as diabetes known to affect gastrointestinal motility [10] and after the chronic treatment with rosiglitazone, an insulin sensitizing agents that shows beneficial gastrointestinal effects [11].

Therefore, in this research we aimed to assess whether the pathway HO/CO could be in some way involved in the constipating effect of a 5-HT_3_ receptor antagonist, such as granisetron. This interaction was investigated both in vitro and in vivo evaluating the effects of an HO inhibitor such as zinc protoporphyrin IX (ZnPPIX) or an HO inducer such as hemin alone or coadministered with granisetron.

## 2. Materials and Methods

### 2.1. Experimental Animal Model

All procedures in animals were performed in accordance with Guidelines and Authorization for the Use of Laboratory Animals (Italian Government, Ministry of Health). 

Male Sprague Dawley rats weighing 220–250 g at arrival (Harlan, San Pietro al Natisone, Udine, Italy) were used. Rats were housed in an animal facility with monitored temperature and light (12 hour cycle and 21 ± 2°C). The animals were allowed to acclimate to the environment for at least 7 days and were then put into individual cages before initiating the study. All rats had free access to water and food when they were not under testing. All animals were handled and trained for at least 1 week to reduce the possible stress induced by drug administration.

### 2.2. Tensiometric Studies

**Figure 1:**
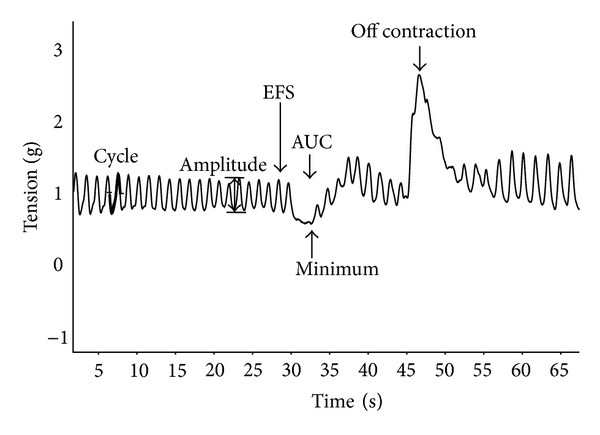
Typical tracings of duodenal longitudinal smooth muscle spontaneous basal activity and inhibitory responses to electrical field stimulation (EFS) (10 Hz; 20 V, 1 msec, pulse trains lasting 30 sec) in control rats.

After general anesthesia (pentobarbital 80 mg/kg/i.p.) rats were killed by cervical dislocation. A 3 cm section of duodenum was then obtained through a midline incision of the abdomen. Specimens (1 cm in length) were immediately placed in a cooled modified Krebs' solution (pH = 7.4) of the following composition (mM): NaCl 113, KCl 4.8, MgSO_4_ 1.2, CaCl_2_ (H_2_O) 2.2, NaH_2_PO_4_ 1.2, NaHCO_3_ 25, glucose 5.5, and ascorbic acid 5.5. Specimens were then cleaned, rinsed, and mounted longitudinally in an organ bath (20 mL) filled with modified Krebs' solution, maintained at 37°C, and aerated with a mixture of 95% O_2_ and 5% CO_2_. One end of the longitudinal duodenum was connected to a metal rod while the other end was attached to a strain gauge transducer (FORT 25, WPI, Sarasota, FL, USA). Isometric tension was measured by the PowerLab data acquisition system and recorded using Chart 5.5.5 (ADInstruments, Castle Hill, Australia). The tissue was allowed to equilibrate for at least 20 minutes prior to the start of the experiment. An initial load of 1.0 g tension was applied to the preparation. The spontaneous basal activity of specimens was recorded. In particular, the number of cycles/15 sec and the mean amplitude of cycle (i.e., mean of differences between the higher point and the lowest point of each cycle in 15 sec-tracing) were evaluated for 15 sec just before electrical field stimulation (EFS) (Figure 1). The specimens were then subjected to transmural stimulation (EFS) at frequencies of 1, 3, 5, 10 Hz (15 V, 1 msec, pulse trains lasting 15 sec) through two parallel platinum electrodes connected to a stimulator (Digital Stimulator, LE 12106, Letica, Ugo Basile, Italy). The EFS induces an immediate relaxation of specimens followed, at the end of EFS, by a contraction called off contraction [12]. Therefore, we evaluated all components of the inhibitory responses obtained after EFS, that is, (a) area under the curve (AUC), expressed as percentage of the maximal effect elicited by sodium nitroprussiate (SNP) since at the end of each evaluation curve, specimens were stimulated with SNP (50 *μ*M); (b) the minimum value of basal tone reached during inhibitory response; (c) the off-contraction (Figure 1). These last two parameters were expressed as percentage of the basal tone recorded immediately before EFS administration. To obtain a nonadrenergic, noncholinergic (NANC) inhibitory response, all experiments were carried out in the presence of atropine (3 *μ*M) and guanethidine (3 *μ*M) [10]. To evaluate the CO component of NANC duodenal motility, spontaneous basal activity and EFS-induced response were measured before and after incubation with ZnPPIX (60 min, 10 *μ*M) or hemin (120 min, 10 *μ*M). The effect of cumulative doses of granisetron (10 min, 0.1, 0.3, and 1 *μ*M) on basal spontaneous activity and EFS-induced response were evaluated alone or after the incubation with ZnPPIX or hemin.

### 2.3. Gastrointestinal Motility Assay

Seventeen hours before the test day, rats were randomly assigned to one of the treatment groups: vehicle treated, granisetron (25, 50, 75 *μ*g/kg/sc), granisetron + ZnPPIX (50 *μ*g/kg/i.p., 60 min before granisetron), and granisetron + hemin (50 *μ*M/kg/i.p. 24 h before granisetron). The timing and dose of ZnPPIX and hemin were carefully chosen in order to obtain the greatest level of HO inhibition or induction, respectively [13, 14]. Rats were then deprived of food with free access to water. On the test day, they were weighed and allowed to free access to food for only 20 min after which the amount of food consumed as well as the weight of rats were recorded. After drug administration, each rat was monitored every 10 min for 180 min. The time to first defecation and number of stool pellets extruded in 180 min were recorded as an index of colonic emptying [15–17]. 

### 2.4. Immunoblotting Detection of HO-1 and HO-2 Isoforms

HO-1 and HO-2 expression was assessed by immunoblotting. Six rats were administered with hemin (50 *μ*M/kg/i.p.) or its vehicle 24 h before sacrifice (after general anesthesia with pentobarbital (80 mg/kg i.p.) rats were killed by cervical dislocation). Duodenal preparations were homogenized and equal amounts of protein (70 *μ*g) were separated by 10% SDS-polyacrylamide gels. After electrophoresis, proteins were transferred to a nitrocellulose membrane and immunoblotted with the following primary antibodies (dilution 1 : 500): HO-1 (Enzo Life Sciences, Farmingdale, NY, USA) and HO-2 (Stressgen Biotechnologies Corp., Victoria, BC, Canada). The β-actin antibody was from Sigma (Chemical Co., St. Louis, MO, USA). Incubation with HRP-conjugated secondary anti-rabbit or anti-mouse antibodies (Amersham, Amersham Biosciences, Little Chalfont, Buckinghamshire, England) (1 : 3000), as appropriate, was performed for 1 h at room temperature. The blot was detected using an enhanced chemiluminescence assay (ECL, Amersham Biosciences, Little Chalfont, Buckinghamshire, England) and visualized by Molecular ChemiDoc XRS System (Bio-Rad Laboratories, Hercules, CA, USA). Images were captured with QuantityOne Software (Bio-Rad Laboratories, Hercules, CA, USA) and immunoblotting results were evaluated by densitometry (Image J, National Institutes of Health, Bethesda, MD, USA).

### 2.5. Drugs and Chemicals

The following drugs were used: atropine sulphate, guanethidine monosulphate, sodium nitroprusside, granisetron, hydrochloride dissolved in saline (Sigma Chemical Co., St. Louis, MO, USA). Zinc protoporphyrin IX and hemin were dissolved in 0.1 N NaOH and aligned to a final pH of 7.4 with HCl (Sigma Chemical Co., St. Louis, MO, USA). Vehicle-treated rats received the same amount of vehicle as drug-treated animals.

### 2.6. Statistical Analysis

Statistical analysis was performed by means of one-way or two-way analysis of variance, as appropriate, followed by *t*-test with Bonferroni's correction or Newman-Keuls multiple comparison test. The level of significance was set at *P* < 0.05. Results are the mean ± S.E.M. from 8–12 preparations for each experiment unless otherwise indicated.

## 3. Results

### 3.1. In Vitro Study

#### 3.1.1. Effect of In Vitro Administration of Granisetron, ZnPPIX, or Hemin and Their Interaction on Spontaneous Basal Activity of Duodenal Preparations

**Figure 2:**
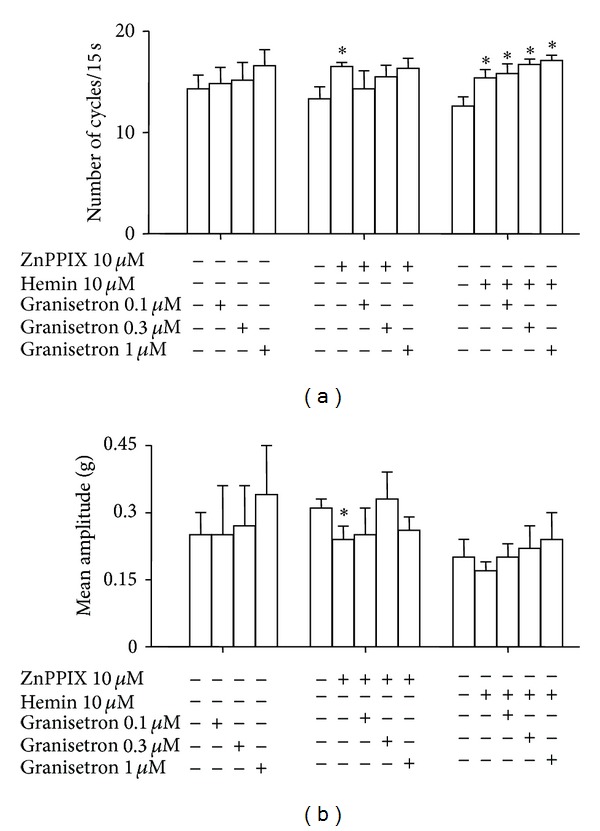
Rat spontaneous basal duodenal activity after ZnPPIX, hemin, or granisetron alone and in combination. (a) ZnPPIX (60 min, 10 *μ*M), an HO-inhibitor, and hemin (120 min, 10 *μ*M), an HO-1 inducer, significantly increase, the number of cycles/15 sec. Granisetron does not affect this parameter. When coadministered, granisetron abolishes ZnPPIX effects but does not influence hemin effect. (b) ZnPPIX significantly reduces mean amplitude. This parameter was not affected by either hemin or granisetron. When coadministered, granisetron abolishes ZnPPIX effect but does not modify the lacking effect of hemin. Values are expressed as mean ± SEM of 8–12 experiments. *P < 0.05 versus their respective control.

Figure 2 shows the effects of ZnPPIX (10 *μ*M), an HO-inhibitor, hemin (10 *μ*M), an HO-1 inducer and granisetron (0.1, 0.3, 1 *μ*M) alone or coadministered on the number of cycles/15 sec and the mean amplitude that characterize the basal spontaneous activity recorded in vitro in duodenal preparations. 

Figure 2(a) shows that ZnPPIX (60 min, 10 *μ*M), an HO-inhibitor, and hemin (120 min, 10 *μ*M), an HO-1 inducer, significantly increase the number of cycles/15 sec. Granisetron does not affect this parameter. When coadministered, granisetron abolishes ZnPPIX effects but does not influence hemin effect.  Figure 2(b) shows that ZnPPIX significantly reduces mean amplitude. This parameter was not affected by either hemin or granisetron. When coadministered, granisetron abolishes ZnPPIX effect but does not modify the lacking effect of hemin. 

In summary, these findings suggest that the inhibition or induction of HO differently affects the basal spontaneous activity of duodenal preparations while granisetron does not modify it. However, granisetron is able to restore spontaneous basal activity when HO activity is inhibited but not when HO activity is increased.

#### 3.1.2. Effect of In Vitro Administration of Granisetron, ZnPPIX, or Hemin and Their Interaction on the Components of EFS-Response of Duodenal Preparations

Table 1 shows that in vitro administration of granisetron, at all doses tested, does not affect any component of response to EFS such as area under the curve, minimum values, and off contraction. 

As previously shown [10, 11], in vitro administration of ZnPPIX (10 *μ*M) significantly reduces the area under the curve (*P* < 0.05) but does not modify the remaining components of EFS response. However, further administration of granisetron (i) abolishes the reducing effect of ZnPPIX on area under the curve thus restoring basal levels (with the exception of the higher dose of granisetron), (ii) does not affect minimum values, and (iii) significantly reduces the off contraction that was unaffected by either in vitro administration of ZnPPIX or granisetron on its own (Table 2).

Hemin administered in vitro significantly increases the area under the curve and decreases the minimum value but does not affect the off contraction. When granisetron is co-administered, the hemin effect on area under the curve and on minimum values (with the exception of the lower dose of granisetron) is reverted with the restoration of basal levels. No effect is recorded on off contraction following administration of either hemin or granisetron alone or in combination (Table 3).

Therefore, we may summarize that inhibition of HO, by ZnPPIX, or increased expression of HO-1, by hemin, differently affect response to EFS. Moreover, in vitro granisetron per se does not affect any component of response to EFS but when HO is inhibited or HO-1 is overexpressed, granisetron either restores the basal levels or adds new effect (decreases off contraction when administered after ZnPPIX).

### 3.2. In Vivo Study

#### 3.2.1. Effect of In Vivo Acute Administration of Granisetron, ZnPPIX, or Hemin and Their Interaction on Transit Time

Rats were homogeneous in their body weight recorded before and after 20 min free access to food on the test day. Moreover, the amount of food eaten before drug administration was comparable between groups (data not shown). 

**Figure 3:**
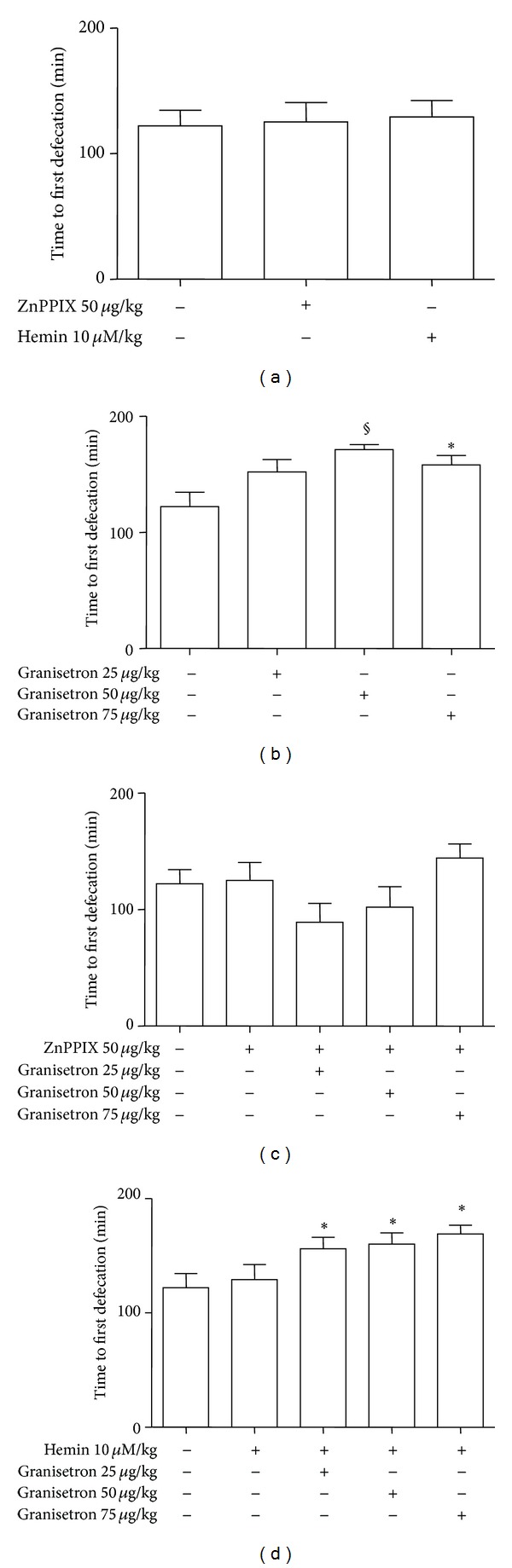
Effect of in vivo administration of ZnPPIX, hemin, or granisetron alone and in combination on the time to first defecation in rats. (a) ZnPPIX and hemin do not affect the time to first defecation. (b) Granisetron significantly increases this parameter at higher doses used. (c) Preadministration of ZnPPIX reduces the time to first defecation in rats treated with granisetron to control levels. (d) Hemin administered before granisetron significantly increases the time to first defecation compared to basal levels. Values are expressed as mean ± SEM of 8–12 experiments. ^§^ *P* < 0.001 and *P < 0.05 versus their respective control.

In vivo administration of ZnPPIX (50 *μ*g/kg) 60 min before 3 hr observation does not affect the time to first defecation. Similarly, hemin administered 24 hr before observation does not alter this parameter (Figure 3(a)). 

In line with the literature data, granisetron (25, 50, 75 *μ*g/kg) has constipating effect: in fact the time to first defecation is significantly increased at higher doses used. In this regard, it can be underlined that the maximum effect is obtained with 50 *μ*g/kg (*P* < 0.001) and not with 75 *μ*g/kg (*P* < 0.05) (Figure 3(b)) suggesting a bell-shaped effect.

Preadministration of ZnPPIX (50 *μ*g/kg) reduces the time to first defecation in rats treated with granisetron to control levels thus abolishing the granisetron constipating effect (Figure 3(c)). Hemin (50 *μ*M/kg) administered 24 hr before granisetron significantly increased the time to first defecation compared to basal levels (*P* < 0.05). Interestingly, hemin cotreatment was able to confer a significant effect to the lowest dose of granisetron (25 *μ*g/kg) that was ineffective per se (Figure 3(d)). We cannot demonstrate an increased efficacy when hemin was coadministered with granisetron at dose of 50 and 75 *μ*g/kg due to ceiling effect; that is, the time to first defecation due to granisetron is so near to the maximum observation time (180 min) that it is very difficult to be able to demonstrate any further increase.

Overall, the constipating effect of granisetron, expressed as increased time to first defecation, is abolished by pre-treatment with ZnPPIX and increased by hemin.

**Figure 4:**
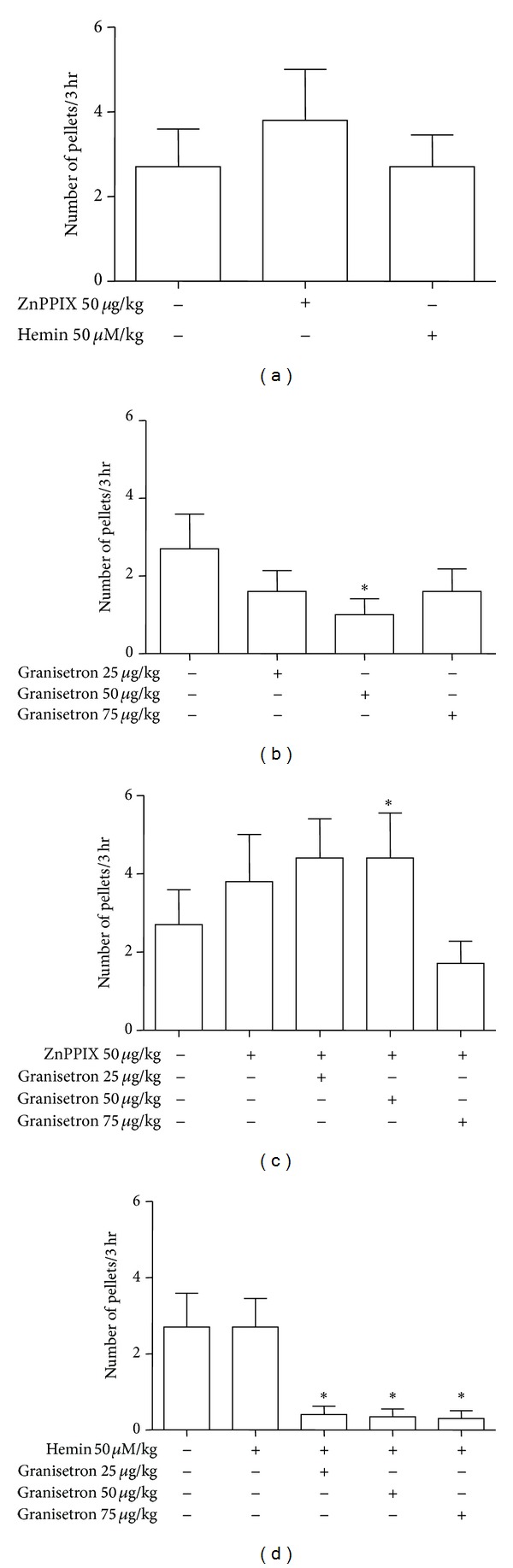
Effect of in vivo administration of ZnPPIX, hemin, or granisetron alone and in combination on number of fecal pellets/3 hr. (a) ZnPPIX and hemin do not affect the number of fecal pellets/3 hr. (b) Granisetron significantly reduces this parameter although only at dose of 50 *μ*g/Kg. (c) Preadministration of ZnPPIX increases the number of fecal pellets/3 hr in rats treated with granisetron to control levels and even higher. (d) Hemin administered before granisetron significantly decreases the number of fecal pellets/3 hr compared to basal levels at all doses of granisetron. Values are expressed as mean ± SEM of 8–12 experiments. *P < 0.05 versus their respective control.

In line with the effect on the time to first defecation, ZnPPIX and hemin do not change the number of fecal pellets collected in 3 hrs of observation (Figure 4(a)). The constipating effect of granisetron can be suggested by the significant reduction of the number of fecal pellets although only at 50 *μ*g/kg suggesting a bell-shaped effect (*P* < 0.05, Figure 3(b)). Interestingly, ZnPPIX significantly increased the number of pellets/3 hr when coadministered with granisetron (50 *μ*g/kg) (*P* < 0.05) thus reverting the granisetron constipating effect (Figure 4(c)). Hemin unmasked the constipated effect of granisetron so that all doses used were able to significantly reduce the number of fecal pellets (*P* < 0.05, Figure 4(d)) compared to basal level. 

In summary, these observations suggest that acute in vivo administration of granisetron alters the transit time so that constipating effects can be seen. In general, these effects are abolished by pre-treatment with ZnPPIX and are increased by hemin. 

### 3.3. Effects of Hemin on HO-1 and HO-2 Protein Expression

**Figure 5:**
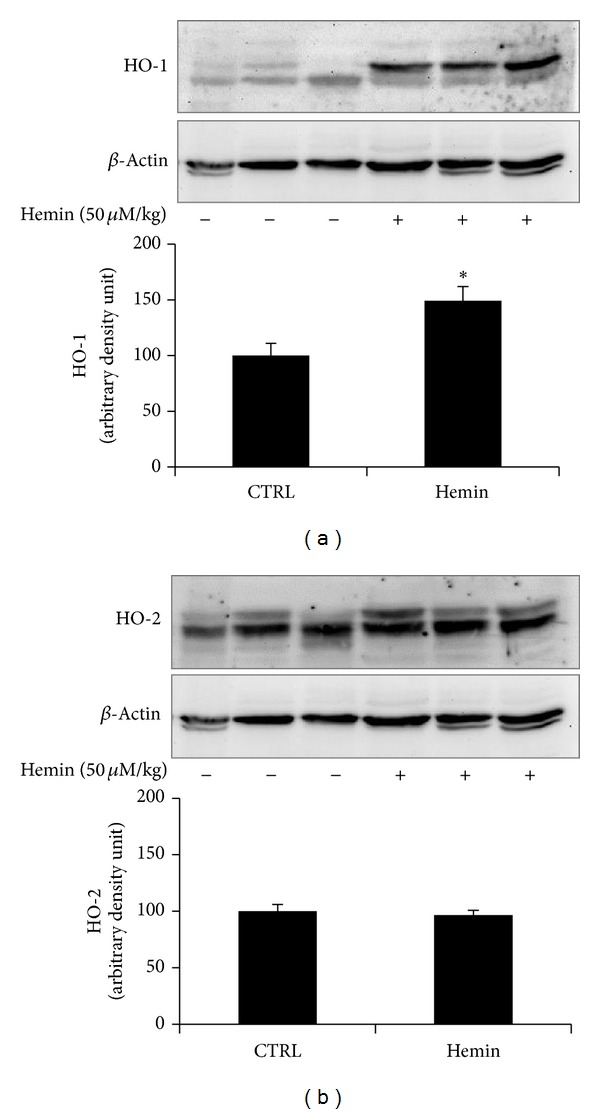
Expression of HO-1 and HO-2 in duodenum from rats treated with hemin. Representative anti-HO-1 (a), anti-HO-2 (b) immunoblots of duodenal homogenates from vehicle- and hemin-treated rats (upper panels), and quantitative densitometry (lower panels). Increased expression of HO-1 was found in hemin- (50 *μ*M/kg)-treated rats compared to control. No difference in HO-2 expression was found among groups. Values are expressed as mean ± SEM of 3 experiments. *P < 0.05 versus WKY (Newman-Keuls multiple comparison test).

Protein levels of HO-1 and HO-2 isoforms were analyzed in tissue homogenates of duodenum from rats treated with hemin (50 *μ*M/kg/i.p) 24 hr before sacrifice. Significant increase in HO-1 protein expression was observed by western blotting analysis between homogenates controls and samples treated with hemin, as shown in Figure 5(a) (*P* < 0.05). Conversely, no significant modification in protein level of constitutive HO-2 isoform was detected neither in controls nor in treated duodenum samples (Figure 5(b)). 

## 4. Discussion

The present study aimed to investigate, both in vivo and in vitro, the possible involvement of HO/CO pathway in the constipating effect of granisetron, a 5-HT_3_ receptor antagonist. 

There are two main results of our research: (a) in vitro granisetron administration alters, although in different ways, the effect of both of ZnPPIX, an HO inhibitor, and hemin, an HO-1 inducer, on some parameters of duodenal motility in NANC conditions; (b) in vivo constipating effect of granisetron is abolished by preatreatment with ZnPPIX and increased by pretreatment with hemin, although ZnPPIX and hemin do not affect gastrointestinal motility per se.

In presence of atropine and guanethidine, that is, in NANC conditions, in vitro incubation with ZnPPIX or hemin differently affects some parameters of spontaneous activity (mean amplitude) as well as EFS-induced response (AUC and the minimum value of inhibitory response to EFS). Neither ZnPPIX nor hemin influences off contraction per se. These findings are in agreement with our previous studies in which we have demonstrated that inhibiting HO (by ZnPPIX) significantly reduces AUC, one component of inhibitory response to EFS [7, 10, 11, 18] and that exogenous administration of CO by a water soluble CO-releasing molecule (CORM-3) increases this component [10]. Moreover, the finding that hemin affects duodenal activity is consistent with a previous study carried out on isolated rabbit jejunum showing a reduction of tone and amplitude of contractions [19]. Overall, present study confirms that, in NANC conditions, HO/CO pathway is involved in duodenal motility. Furthermore, the effect of ZnPPIX may suggest that the products of HO activity (among which carbon monoxide) are tonically released and contribute to modulate duodenal motility.

In vitro experiments indicate that granisetron does not affect either the parameters of spontaneous duodenal activity or the EFS-induced response when administered in NANC conditions. These findings are in agreement with those of other investigators that suggest only a modulatory effect of 5-HT_3_ receptors rather than a direct involvement in peristalsis [20]. 

When granisetron was administered in vitro to duodenal preparations pretreated with ZnPPIX, it was able to abolish the effect of ZnPPIX on spontaneous motility and on EFS-induced response. The preincubation with hemin followed by granisetron resulted in preserving the effect of hemin on spontaneous basal activity and in abolishing the effect on the AUC and minimum value of EFS-induced response. Overall, the components of both spontaneous activity and EFS-induced response are differently affected by the coadministrations of ZnPPIX or hemin with granisetron, in line with in vivo studies (see below).

In vivo studies showed no effect of ZnPPIX or hemin on gastrointestinal transit as indicated by the lack of effects on both time to first defecation and number of fecal pellets/3 hr. Similar results have been reported in a previous study showing that hemin did not affect intestinal transit when administered to sham-operated rats with respect to rats with burn injury [14]. Moreover, systemic administration of hemin or ZnPPIX alone had no effect on intestinal transit in control rats in studies that demonstrated a protection by hemin against LPS-induced septic ileus [21]. 

However, the effects of ZnPPIX and hemin obtained in our experiments in vitro do not overlap with those obtained in vivo. In this regard, it has to be underlined that in vitro studies were carried out in NANC conditions, that is, abolishing the cholinergic and adrenergic components of intestinal activity. Those components were intact in in vivo study. This discrepancy may suggest that the role of HO/CO components of intestinal activity is not predominant and that more represented components (cholinergic and adrenergic) need to be kept out in order to emphasize those players. However, the role of HO/CO components of intestinal activity can be unmasked in vivo when 5-HT_3_ receptors are blocked (see below). 

Acute in vivo administration of granisetron significantly reduced gastrointestinal transit in agreement with the literature data that report constipation as one of the most represented side effects when the drug is used as antiemetic [22–24]. It has also been shown that postabdominal irradiation treatment with granisetron is able to significantly decrease the diarrhoea score in rat [14]. 

Interestingly and in line with in vitro data, an interactive effect was seen when granisetron was administered after ZnPPIX or hemin. Pretreatment with ZnPPIX overall altered granisetron constipating effect: in fact, cotreatment abolishes the increase of the time to defecation and revert the decrease of the number of pellets/3 hr. Hemin pretreatment clearly increases the effect of granisetron alone so that this drug affected both parameters of intestinal transit even at dose levels inactive when administered alone.

To the best of our knowledge, this is the first study reporting an interaction between HO/CO pathways and an antagonist of 5-HT_3_ receptors at gastrointestinal level. In fact, the only study that has demonstrated the possible interplay between 5-HT_3_ receptors and carbon monoxide, a by-product of HO activity, has been carried out in cell culture from CNS such as superior cervical ganglion [25]. 

Although our data obtained in vitro suggest that this interaction is present peripherally (i.e., at duodenal level), systemic administration of drugs does not allow us to rule out central effects of hemin and granisetron (ZnPPIX is blood-brain barrier impermeant). In fact, it has been shown that 5-HT_3_ receptor antagonists block serotonin binding at vagal afferents in the gut and in the regions of the CNS involved in emesis [26] but have also anxiolytic and antipsychotic effect or show some effect in migraine [22]. Moreover, systemic administration of hemin may induce HO-1 in CNS since attenuation of hippocampal injury in rats after acute carbon monoxide poisoning through induction of HO-1 was shown [27]. 

Our in vivo results are not in agreement with the literature data as studies carried out in mice with targeted deletions of HO-2 showed reduced in vitro ileal relaxation and in vivo significantly slower overall transit [7]. However, in our study the prokinetic effect of ZnPPIX, which reduces HO activity, and the constipating effect of hemin, which induces increased expression of HO-1, are seen only when coadministered with granisetron. 

Therefore, some considerations are possible: (1) every time a reduced or increased levels of HO/CO are present it is possible that granisetron will lose or increase its efficacy; (2) in order to modulate the effect of granisetron, in either ways, it is possible to act on HO/CO pathway.

HO-1 has drawn much attention as a molecule with potent antioxidant, antiinflammatory, and anti-proliferative effects and several studies suggest that HO could be the target for drugs aimed to treat a high variety of diseases from cardiovascular disease to obesity [28–30], from organ transplantation to gastrointestinal affections [31–34]. In this regard, systemic administration of hemin has been proposed to treat gastroparesis of different origins [35]. Pegylated zinc protoporphyrin, a potent HO inhibitor, administered in vitro induced apoptosis of human colon carcinoma SW480 cells and inhibited growth of murine colon carcinoma in vivo [36]. Therefore, our findings suggest that future treatment able to increase or decrease HO activity at gastrointestinal level will have to take into account the possible interaction with other drugs such as granisetron. Moreover, it has to be considered that several pathological situations are characterized by increased expression of HO-1 such as human gastric cancer [37], systemic juvenile idiopathic arthritis [38], and acute respiratory distress syndrome [39].

As far as the second series of considerations, our study may indicate a possible therapeutic synergistic use of granisetron plus agents able to reduce HO activity to counteract its constipating effect or plus agents able to induce HO to increase its activity when a reduced intestinal motility is required. 

In conclusion, granisetron may have more or less pronounced effects depending on the induced or previous level of HO activity: this could be of interest since the HO involvement in several pathophysiological situations and since the possible trend to develop drugs that will affect its activity.

## Figures and Tables

**Table 1:** Effect of in vitro administration of granisetron (G) on the components of EFS response of duodenal preparations.

Treatments (*μ*m)	Area under the curve (% SNP)			*P*	Minimum value (% mean basal tone)			*P*	Off contraction (% mean basal tone)			*P*
	3 Hz	5 Hz	10 Hz		3 Hz	5 Hz	10 Hz		3 Hz	5 Hz	10 Hz	
Basal	23.7 ± 6.8	29.3 ± 7.8	32.2 ± 10.4		75.8 ± 5.7	72.7 ± 6.6	69.3 ± 7.1		151.1 ± 19.0	154.1 ± 10.9	190.9 ± 23.7	
G 0.1	29.5 ± 8.6	33.9 ± 8.2	35.3 ± 8.7	n.s.	72.4 ± 6.1	70.0 ± 6.2	66.2 ± 7.1	n.s.	148.3 ± 13.8	159.8 ± 10.5	193.8 ± 26.9	n.s.
G 0.3	27.8 ± 7.1	27.7 ± 9.3	31.7 ± 9.1	n.s.	72.2 ± 6.5	72.4 ± 7.7	73.2 ± 8.3	n.s.	145.1 ± 14.7	154.5 ± 14.4	209.1 ± 22.1	n.s.
G 1	35.7 ± 6.9	37.8 ± 8.2	42.1 ± 7.3	n.s.	56.6 ± 11.5	63.2 ± 7.0	57.5 ± 7.0	n.s.	156.0 ± 14.3	169.9 ± 18.4	210.6 ± 32.3	n.s.

**Table 2:** Effect of in vitro administration of ZnPPIX alone or in combination with granisetron (G) on the components of EFS response of duodenal preparations.

Treatments (*μ*m)	Area under the curve (% SNP)			*P*	Minimum value (% mean basal tone)			*P*	Off contraction (% mean basal tone)			*P*
	3 Hz	5 Hz	10 Hz		3 Hz	5 Hz	10 Hz		3 Hz	5 Hz	10 Hz	
Basal	44.4 ± 5.1	51.8 ± 8.1	54.9 ± 3.4		61.04 ± 5.3	52.4 ± 5.1	49.1 ± 7.2		172.6 ± 10.2	174.9 ± 10.1	228.1 ± 12.4	
ZnPPIX 10	35.4 ± 4	41.8 ± 4.8	44.3 ± 3.9	0.05	59.2 ± 5.2	52.5 ± 6.5	46.4 ± 5.4	n.s.	179.8 ± 19.1	202.8 ± 22.9	261.9 ± 35.9	n.s.
ZnPPIX + G 0.1	35.8 ± 5.5	39.5 ± 4.8	44.7 ± 5.3	n.s.	65.6 ± 5.9	59.4 ± 6.5	53.5 ± 8.1	n.s.	158.6 ± 13.8	179.1 ± 11.6	185.9 ± 26.8	0.05
ZnPPIX + G 0.3	37.8 ± 4.5	42.7 ± 4.6	47.0 ± 6.1	n.s.	59.3 ± 5.8	59.7 ± 5.1	55.1 ± 5.4	n.s.	138.7 ± 21.8	171.6 ± 9.9	206.8 ± 12.2	0.05
ZnPPIX + G 1	31.3 ± 4.2	33.3 ± 4.6	35.2 ± 6.3	0.05	61.8 ± 4.8	64.1 ± 4.8	54.9 ± 7.1	n.s.	155.1 ± 5.6	173.0 ± 8.9	203.9 ± 19.1	0.05

**Table 3:** Effect of in vitro administration of hemin alone or in combination with granisetron (G) on the components of EFS response of duodenal preparations.

Treatments (*μ*M)	Area under the curve (% SNP)			*P*	Minimum value (% mean basal tone)			*P*	Off contraction (% mean basal tone)			*P*
	3 Hz	5 Hz	10 Hz		3 Hz	5 Hz	10 Hz		3 Hz	5 Hz	10 Hz	
Basal	28.4 ± 4.1	34.2 ± 3.8	43.8 ± 4.5		72.1 ± 4.3	62.64 ± 4.3	54.7 ± 5.3		133.8 ± 6.6	144.7 ± 7.8	178.2 ± 16.4	
Hemin 10	33.6 ± 4.4	40.0 ± 4.2	49.4 ± 4.4	0.05	61.9 ± 4.5	58.7 ± 4.6	51.9 ± 5.9	0.05	138.0 ± 6.4	153.9 ± 8.5	185.8 ± 11.3	n.s.
Hemin + G 0.1	29.8 ± 3.6	36.2 ± 3.3	46.3 ± 3.6	n.s.	62.2 ± 4.4	57.5 ± 4.8	50.2 ± 5.7	0.05	146.7 ± 8.2	157.2 ± 9.9	200.7 ± 13.2	n.s.
Hemin + G 0.3	26.1 ± 3.2	33.8 ± 3.5	40.0 ± 3.1	n.s.	60.4 ± 5.1	55.5 ± 6.4	49.1 ± 7.4	n.s.	141.6 ± 6.2	156.4 ± 9.4	211.8 ± 26.8	n.s.
Hemin + G 1	23.6 ± 2.8	28.2 ± 3.2	34.3 ± 3.2	n.s.	59.5 ± 7.3	60.1 ± 6.4	55.3 ± 6.6	n.s.	158.2 ± 12.7	166.6 ± 14.2	195.8 ± 14.8	n.s.
